# DISPARE: DIScriminative PAttern REfinement for Position Weight Matrices

**DOI:** 10.1186/1471-2105-10-388

**Published:** 2009-11-26

**Authors:** Isabelle da Piedade, Man-Hung Eric Tang, Olivier Elemento

**Affiliations:** 1 Bioinformatics Centre, Department of Biology & Biotech Research and Innovation Centre, University of Copenhagen, Ole Maaløes Vej 5, DK-2200 Copenhagen N, Denmark; 2 Institute for Computational Biomedicine Weill Medical College of Cornell University, New York, NY, USA

## Abstract

**Background:**

The accurate determination of transcription factor binding affinities is an important problem in biology and key to understanding the gene regulation process. Position weight matrices are commonly used to represent the binding properties of transcription factor binding sites but suffer from low information content and a large number of false matches in the genome. We describe a novel algorithm for the refinement of position weight matrices representing transcription factor binding sites based on experimental data, including ChIP-chip analyses. We present an iterative weight matrix optimization method that is more accurate in distinguishing true transcription factor binding sites from a negative control set. The initial position weight matrix comes from JASPAR, TRANSFAC or other sources. The main new features are the discriminative nature of the method and matrix width and length optimization.

**Results:**

The algorithm was applied to the increasing collection of known transcription factor binding sites obtained from ChIP-chip experiments. The results show that our algorithm significantly improves the sensitivity and specificity of matrix models for identifying transcription factor binding sites.

**Conclusion:**

When the transcription factor is known, it is more appropriate to use a discriminative approach such as the one presented here to derive its transcription factor-DNA binding properties starting with a matrix, as opposed to performing *de novo *motif discovery. Generating more accurate position weight matrices will ultimately contribute to a better understanding of eukaryotic transcriptional regulation, and could potentially offer a better alternative to *ab initio *motif discovery.

## Background

Gene expression is controlled by the interaction of transcription factors (TFs) and their DNA binding sites. Promoters often contain multiple binding sites for different TFs that work in tandem to control the regulation (activation or inhibition) of gene expression. TFs bind to short (typically 6-14 bp) degenerate DNA sequence patterns or motifs, which makes them relatively difficult to find. Understanding the regulation of gene expression in higher eukaryotes is still a major challenge and the current algorithms are not always able to clearly distinguish real transcription factor binding sites (TFBS) from random look-alike sequences [1,2].

Position-weight matrices (PWMs) are widely used to represent TFBS in promoter regions of genes [2-4]. Unfortunately, many of these PWMs have low information content and match a huge number of sequences in the genome. However, most of these matches are likely to be false positives [5,6]. PWMs are commonly used due to lack of better alternatives [7,8]. PWMs are calculated from position frequency matrices (PFMs) that contain the observed nucleotide frequencies at each position in the profile alignment of binding sites for a TF of interest. Most of the known matrix models for TFBS can be obtained from the JASPAR [9] and TRANSFAC [10] databases, together with the set of binding sites used to build them. TFBS based on DNase I footprinting experiments can be found in the DNAse I database for *Drosophila melanogaster *[11]. Furthermore, the matrices are often derived from a limited number of experimentally verified sequences, which leads to inaccurate representations of TF-DNA binding affinities. Today, wet-lab technologies such as chromatin immunoprecipitation (ChIP) [12] are commonly used to identify in vivo TF binding locations. ChIP-chip and ChIP-PET analyses provide large amounts of data and will therefore pave the way for the construction of more precise PWMs. In particular, these experiments provide an opportunity to computationally improve the accuracy of existing position-specific matrices. It is this important problem that we address in the present study.

## Results and Discussion

We have created DISPARE, a two-step algorithm that takes a known matrix as its input, performs an iterative and discriminative refinement using experimentally verified TFBS and background sequences. After the discriminative optimization step, DISPARE returns an optimized matrix with improved information referred to as a DISPARE matrix. Using the width and phase optimization step, a width-optimized matrix is obtained with improved information and optimized width and phase (see Methods and Additional file 1: Figure S1). In order to evaluate the performance of our algorithm, we applied it to several synthetic datasets. We then tested DISPARE using mammalian transcription factor binding sites from experimental ChIP-PET data: human p53 motif from [13], mouse Nanog [14] and human estrogen receptor *α *binding sites [15].

### Synthetic Data

**Figure 1:**
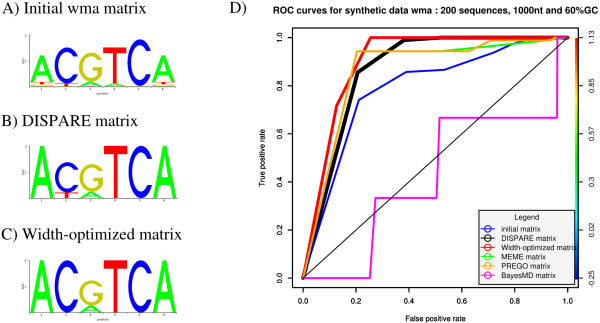
**Refinement of the synthetic matrix wma**. The sequence logos of the initial, DISPARE and width-optimized matrices are shown in A), B) and C). We compared the performances of these three reference models against four motif discovery tools: BayesMD, MEME, PREGO and WEEDER. The ROC performances of this assessment are summarized in D). The DISPARE discriminative refinement algorithm improved the initial matrix and obtained better results that the other methods.

We first applied DISPARE to synthetic data to evaluate its performance under known conditions. A motif was artificially implanted in each of the randomly generated sequences at a random position. The implanted pattern is described by a weight matrix *wma *of width 6 (Figure 1A) and characterized by the consensus 'ACGTCA'. We arbitrarily used the MA0129 JASPAR matrix as the initial matrix. We generated a set of 400 random, 1000 nucleotide-long sequences as a positive set using decodeanhmm [16], a HMM-based random sequence generator. The order of the Markov model was chosen to be equal to 0 so that all nucleotides in the generated sequences were independent of each other. These sequences were GC-rich, containing 30% G, 30% C, 20% A and 20% T. The generated sequence set was divided into a training set and a test set of equal sizes, i.e of 200 sequences each. We copied and shuffled the 400 randomly generated sequences to obtain two corresponding background sets of 200 sequences each.

We ran DISPARE on the positive and background sequences, using the matrix *wma *as input (Figure 1A). The algorithm first performs an iterative matrix optimization by calculating cut-offs that discriminate best between the two datasets. After running the DISPARE discriminative optimization step for 2 iterations, we obtained a DISPARE matrix (Figure 1B). After the first optimization step, the algorithm performs width and phase optimization on the DISPARE matrix in order to investigate whether a better width can be found for the input matrix. This step can improve the accuracy of PWMs because initial matrices often have sub-optimal length and phase. The phase can be defined as position of the window, relatively to the actual pattern on the DNA. In this example, since the PWM was already in its optimal form, the length and phase remained unchanged. The width-optimized matrix thus obtained was 6 nucleotides long (Figure 1C).

We compared the performances of the initial matrix, DISPARE matrix and width-optimized matrix to four PWMs obtained with four *de novo *motif discovery programs: BayesMD, MEME, PREGO and WEEDER (see Methods). The ROC curves of this assessment are illustrated in Figure 1D. We ran MEME using the consensus 'ACGTCA' as prior information so that we could compare our program with another supervised approach.

DISPARE improved the initial matrix by increasing the AUC from 0.78 to 0.87 and outperformed BayesMD (AUC of 0.42). However, MEME achieved the same result as the DISPARE matrix under supervision with the consensus 'ACGTCA' as prior information (AUC of 0.86). *K*-mer based methods gave different results: PREGO was able to find the motif *wma*, achieving an AUC of 0.86, while WEEDER failed to recover the motif *wma *which is why an ROC curve for this tool is omitted in Figure 1D. The width-optimized matrix (AUC of 0.90) improved the performance of the DISPARE matrix due to the additional information enhancement step provided by the width optimization algorithm after the choice of the optimal frame (see Methods).

#### Test of robustness using two synthetic matrices

An important challenge in motif finding is to design methods that can accommodate datasets of various sizes and background compositions. In order to evaluate the robustness of DISPARE in this context, we constructed five new datasets (Table 1) using the initial synthetic matrix *wma*. We increased the number of sequences to 400 (200 training sequences and 200 test sequences) from 1000 (500 training sequences and 500 test sequences) while keeping the length of sequences constant at 1000 nucleotides and the same composition of nucleotides (60%GC) in set 1. The difference between set 1 and set 2 is the increase in the length of the sequences to 2000 nucleotides. Set 3 has the same size as the dataset used in Figure 1 (400 sequences) but has a different background composition (50% GC instead of 60% GC). For sets 4 and 5, we generated datasets with 400 sequences of 10000 nucleotides with two different background compositions. As described above, we divided each set of newly generated sequences into a test set and a training set of equal sizes i.e of 200 sequences each.

**Table 1:** Different sets of matrix wma

Different sets of matrix *wma*			
Set 1	1000 sequences (500 test + 500 training) and 2 × 500 background sequences	1000 nucleotides	60% GC

Set 2	1000 sequences (500 test + 500 training) and 2 × 500 background sequences	2000 nucleotides	60% GC

Set 3	400 sequences (200 test + 200 training) and 2 × 200 background sequences	1000 nucleotides	50% GC

Set 4	400 sequences (200 test + 200 training) and 2 × 200 background sequences	10000 nucleotides	60% GC

Set 5	400 sequences (200 test + 200 training) and 2 × 200 background sequences	10000 nucleotides	50% GC

We ran DISPARE and the four selected motif discovery programs on these five datasets and obtained ROC curves illustrated in Additional file 2: Figure S2. As before, MEME was run using a supervised mode with the consensus 'ACGTCA' as prior information. Running DISPARE on test set 1, which contained a higher number of sequences (500 instead of 200, see Additional file 2: Figure S2), improved the initial matrix once again (AUC of 0.78) by increasing the AUC of both DISPARE matrix and width-optimized matrix to 0.84 and performed better than PREGO (AUC of 0.64). MEME (AUC of 0.83 under supervision) achieved a similar result by accurately capturing the motif *wma*. We did not find any suitable motifs for this set using BayesMD and WEEDER despite several runs. Encouraged by this result, we increased the sequence length in set 2 (Additional file 2: Figure S2) to check the impact of varying sequence lengths on the algorithm. In this test, the DISPARE matrix (AUC of 0.75) and the width-optimized matrix (AUC of 0.75) improved the initial matrix (AUC of 0.71). They performed better than PREGO matrix (AUC of 0.69). However, MEME with supervision achieved a better result (AUC of 0.79). Again, BayesMD and WEEDER failed to recover the 6 nucleotide-long motif. In the third test, (Additional file 2: Figure S2), we investigated the effects of a change in the background composition on the results. Again, our algorithm recovered the motif *wma *in this test (AUC of 0.84 for the DISPARE and width-optimized matrix compared to 0.80 for PREGO, 0.79 for MEME under supervision mode and 0.77 for the initial matrix, 0.63 for BayesMD), showing that changing the background, in which the sites were implanted, had no impact on the accuracy of DISPARE. Finally, we compared the behaviour of each program using random sequences of length 10000. Sets 4 and 5 (Additional file 2: Figure S2) were designed to produce a strong pattern-drowning effect for motif *wma*, as such a short and 'random' pattern (consensus 'ACGTCA') was likely to occur in several locations by chance. In test 4, using a GC-rich background, we showed that DISPARE (AUC of 0.53), MEME and BayesMD (AUC of 0.53) successfully recovered the motif *wma*, while PREGO (AUC of 0.33) and WEEDER failed to capture the correct consensus. Finally, in test 5, using a uniform background (50% GC), we found that only DISPARE, MEME and PREGO were able to capture the motif with a low sensitivity (AUC of 0.54). This low value can be interpreted as the 'baseline' prediction rate for the *wma *matrix for this dataset (AUC of 0.54). These last two tests showed that our discriminative approach is robust against noise, accurately returning a refined matrix from large datasets.

In these five tests, we showed that DISPARE consistently improved the initial matrix and achieved one of the two best ROC performances each time. The width optimization step did not yield further improvements since the initial matrix (6 nucleotides long) was already in its most compact form. Interestingly, we found that for sets larger than 200 sequences, BayesMD and WEEDER failed the test, despite several runs to find a matrix. This can be explained by the 'randomness' of the matrix *wma *consensus or by the statistical background models used in these two approaches which do not recapitulate the background sequences accurately. PREGO, which uses the actual background sequences as input, was able to recover the planted motif. Similarly, MEME uses a 0^th ^order background in its default settings which seemed to work better in this assessment than BayesMD and WEEDER which use higher order backgrounds by default.

After evaluating DISPARE using five different datasets for the matrix *wma*, we re-tested it against a new synthetic matrix. We used the matrix *wm *to generate five new positive and background datasets (Table 2) and changed parameters such as the number of sequences, the background composition and the length of the sequences. The synthetic *wm *motif is 14 nucleotides long and contains the central pattern 'ACGTGC'.

**Table 2:** Different sets of matrix wm

Different sets of matrix *wm*			
Set A	1000 sequences (500 test + 500 training) and 2 × 500 background sequences	1000 nucleotides	60% GC

Set B	1000 sequences (500 test + 500 training) and 2 × 500 background sequences	2000 nucleotides	60% GC

Set C	400 sequences (200 test + 200 training) and 2 × 200 background sequences	1000 nucleotides	50% GC

Set D	400 sequences (200 test + 200 training) and 2 × 200 background sequences	10000 nucleotides	60% GC

Set E	400 sequences (200 test + 200 training) and 2 × 200 background sequences	10000 nucleotides	50% GC

As described above, we divided each set into a training set and a test set, on which the assessments were performed. We generated a corresponding set of background sequences by shuffling each set of positive sequences. The ROC curves of these five tests are shown in Additional file 3: Figure S3. We ran the *de novo *motif finding program MEME using the consensus 'TACGTGCG' as prior information so that we could compare DISPARE with another program running in supervised mode. In test A, we ran DISPARE on a set of 500 sequences, each being 1000 nucleotides long, with a GC-rich background. The initial matrix, the DISPARE matrix and the width-optimized matrix obtained similar results (AUC between 0.90 and 0.92) and did better than the four motif discovery methods (MEME matrix: AUC of 0.88, PREGO matrix: AUC of 0.81, BayesMD matrix: AUC of 0.86, WEEDER matrix: AUC of 0.87). In test B, we increased the sequence length to 2000 nucleotides and compared it with the set A. Again, the initial matrix, the DISPARE matrix and the width-optimized matrix obtained the best AUC (0.88). More interestingly, we found that three tested motif discovery tools could not find the matrix *wm *accurately (PREGO matrix: AUC of 0.39, BayesMD matrix: AUC of 0.49, WEEDER matrix: AUC of 0.59). MEME, however, which used a 0^th ^order background and prior information was able to find the motif *wm *in this configuration (AUC of 0.80). This test demonstrates that DISPARE is robust against pattern drowning and performs accurately with increasing sequence length and the number of sequences.

We then wondered whether background sequence composition could affect the performance of our matrix refinement algorithm. In test C, we constructed a dataset of 200 sequences spanning 1000 nucleotides and containing equal proportion (25%) of each nucleotide. The DISPARE matrix (AUC of 0.92), the width-optimized matrix (AUC of 0.92) and the initial matrix (AUC of 0.92) obtained similar results and performed better than other approaches (MEME matrix under supervised mode: AUC of 0.84, PREGO matrix: AUC of 0.82, BayesMD matrix: AUC of 0.84, WEEDER matrix: AUC of 0.74). This test showed that background composition had no impact on the accuracy of DISPARE.

Finally, in tests D and E (Additional file 3: Figure S3) we evaluated DISPARE and the four selected programs under the extreme situation of looking for the motif *wm *in 10 kb long input sequences. The sequence sets contain 200 sequences but the background composition is GC-rich (test D) and uniform (test E). In test D, the ROC curves show a global decrease of accuracy compared to the test with shorter sequences. However, the DISPARE matrix (AUC of 0.76) and the width-optimized matrix (AUC of 0.75) recovered the initial model (AUC of 0.77). DISPARE performed better than other programs in test D (PREGO matrix: AUC of 0.11, WEEDER matrix: AUC of 0.59), MEME (AUC of 0.14) and BayesMD. We repeated the test with a different background (Additional file 3: Figure S3). The initial matrix, DISPARE matrix and width-optimized matrix obtained consistently similar outcomes (between 0.82 and 0.83). We observed that other methods performed better than in test D (PREGO matrix: AUC of 0.54, BayesMD matrix: AUC of 0.72, WEEDER matrix: AUC of 0.75, MEME matrix under supervised mode: AUC of 0.55).

Increasing the number of sequences (test A) or using longer sequences (tests B and D) had no impact on the performance of DISPARE, unlike motif discovery methods which can sometimes suffer from pattern drowning, i.e. lose the ability to capture the motif signal when the latter is embedded in large amounts of random sequences. Furthermore, we also obtained similar results while running DISPARE on background sequences with a different composition (tests C and E with 50% GC). The performance gain in these tests was expected to be small since the input matrix was the same as the one that we used to build the five datasets. The purpose of this assessment was to evaluate the robustness of DISPARE under various operating conditions.

The tests on synthetic data showed that DISPARE was able to improve the width and information of an initial matrix using sequences containing sites described by the same matrix. In the next test, to assess the performance of DISPARE under more realistic conditions, we distorted the initial synthetic matrix *wm *and ran the assessment again on the same datasets.

#### Data distortion

**Figure 2:**
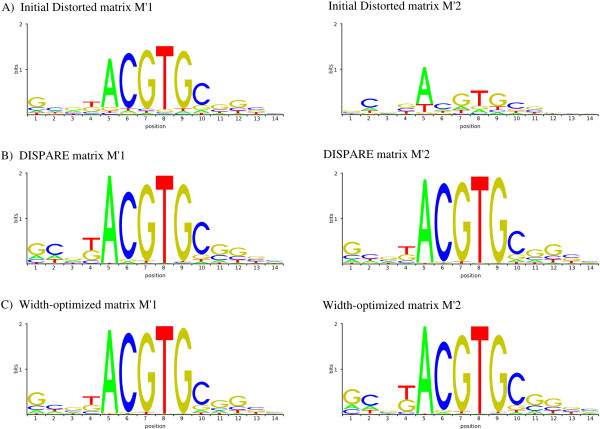
**Refinement of distorted matrices**. The sequence logos of two matrices with weak and strong level of distortion are shown in sub-figure A. Using sequence datasets containing non-distorted binding sites we recovered successfully the initial matrix with DISPARE (sub-figure B). Sub-figure C shows the output of the frame optimization step of our algorithm.

We tested the ability of DISPARE to improve a matrix with distorted information using high quality transcription factor binding sites provided by a positive set. Using the initial synthetic data matrix *wm *(also called *M*) we generated two distorted matrices,  and  (Figure 2A), with the same width as the initial matrix *wm*. This initial synthetic matrix is 14 nucleotides long and consists of the core motif 'ACGTGC' in the center. Uniformly distributed (*U*) noise was added to the matrix *M *to create the elements of *M'*. The elements of the noise matrix *U *are uniformly distributed random numbers between 0 and 1. A constant *k *was used to set the amplitude of noise. We used a constant (*k *= 1) to obtain the first matrix  and used a larger constant (*k *= 10) to obtain a second matrix  with more distortion. *M' *can therefore be expressed as *M' *= *M*+k*U*. The distorted matrices are normalized in a column-wise manner before being returned in order to obtain correct position frequency matrices. We calculated the degree of data distortion from  to *M *and from  to *M*. First, we measured the signal attenuation after distorting the matrices with noise.

The signal attenuation or loss of information content (IC) is defined here by the log-ratio of the information content of the original to the distorted matrix, expressed in decibels (dB):(1)

For matrix , the information content was attenuated by 1.14 dB (from 12.14 to 9.32) and for matrix , the attenuation was equal to 4.85 dB (from 12.14 to 3.98), implying that  signal strength is twice as less as . We then evaluated the data distortion by calculating the difference of Frobenius norm. The Frobenius norm is defined as(2)

It is often used in linear algebra to measure the distortion of a matrix as it is proportional to the mean squares of the errors (see example in [17]). The relative value difference (VD) of the Frobenius norm represents the relative error between the elements of the original and distorted matrices:(5)

where *V D *is the difference of the Frobenius norm from *M' *to *M *divided by the Frobenius norm of *M*. *V D *values for  and  were respectively *V D*_1 _= 0.07 and *V D*_2 _= 0.22.

 is lightly attenuated and distorted, and therefore mimics the case when the count matrix was built with good quality sites (i.e containing a low fraction of non-binding sequences).  mimics the case when sites come from either computational prediction or from low quality experimental sequences (large number of false positive sequences).

**Figure 3:**
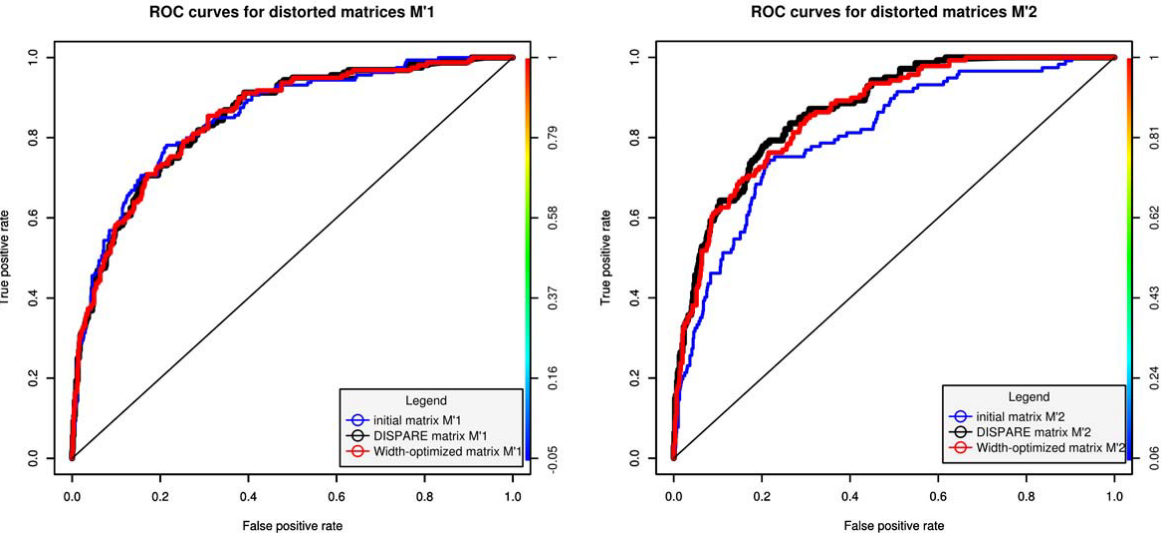
**ROC curves for distorted matrices  and **. The performance improvement seen in the ROC curves for the DISPARE matrix compared to the initial one shows that our algorithm can recover the binding specificities of a distorted model using a set of high quality binding sites.

In order to determine if DISPARE could improve low quality matrices using sequences containing high quality binding sites, we ran our algorithm on the datasets with the distorted matrices. The program converged after 2 iterations using  and 3 iterations using . The DISPARE matrix and the width-optimized matrix recovered the motif core 'ACGTGC' for  and  (Figure 2B and Figure 2C). To measure the performance again after refinement of the distorted matrices, we generated the ROC curves of  and  before and after the discriminative optimization step (Figure 3). We did not increase the AUC in both cases. The performance of the initial matrix (AUC of 0.85), the DISPARE matrix (AUC of 0.85) and the width-optimized matrix (AUC of 0.85) did not change for . However, as we used a more distorted matrix , we got different results. The increase of the AUC from the initial matrix (AUC of 0.81) to the DISPARE matrix (AUC of 0.87) and to the width-optimized matrix (AUC of 0.86) for  shows a gain in the sensitivity and specificity in the whole ROC space from the initial matrix to the DISPARE and the width-optimized matrices. Furthermore, we saw no improvement in our test using  as input since this matrix was slightly distorted compared to the reference sites contained in the datasets. We demonstrated that DISPARE improved the model significantly in the case of a strongly distorted matrix like .

We also measured the gain in information content after width optimization step. Before optimization, the values of the information content of the initial matrices  and  were respectively equal to 9.32 and 3.97. After running DISPARE, we improved these values to 12.20 and 12.66. Regardless of the degree of distortion, DISPARE was able to improve the matrices using the information from high quality sequence data, providing width-optimized matrices with stronger information content. The test on distorted data demonstrated that our algorithm was able to recover and refine matrices with low information content using good quality reference binding sites. In the following test, we perform a similar assessment by refining known matrix models using three different ChIP-PET datasets.

### ChIP-PET data

#### Human p53

**Figure 4:**
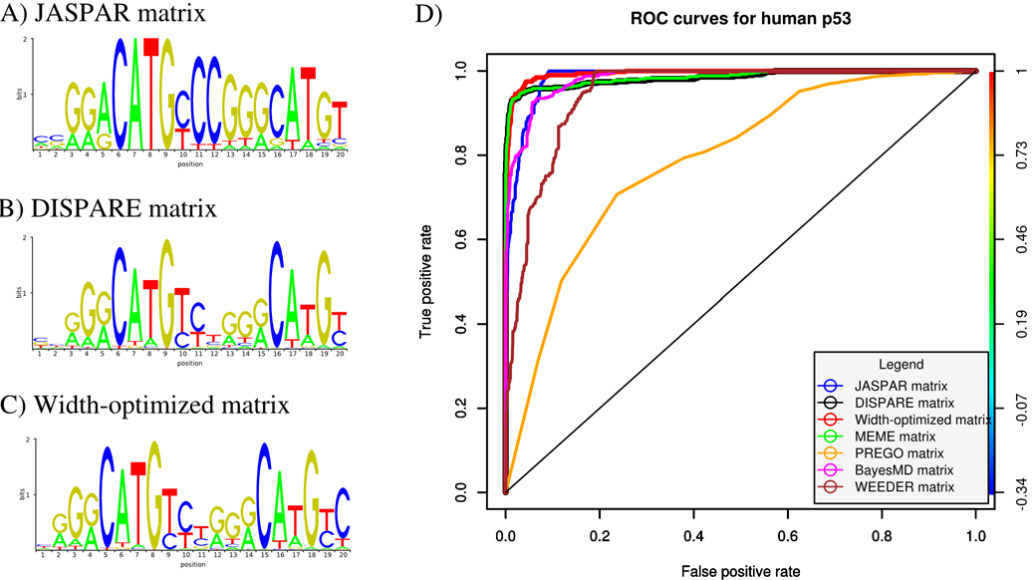
**Refinement of the p53 matrix (JASPAR)**. The sequence logos of the initial JASPAR human p53, DISPARE and width-optimized matrices are shown in A), B) and C). The performances of these three models are compared to those of four motif discovery programs: BayesMD, MEME, PREGO and WEEDER. The results are summarized in the ROC curves shown in D).

p53 is a tumor suppressor protein encoded by p53 gene and binds to DNA in the cells. Due to its role in normal cells as the 'guardian of the genome', it is believed that p53 is inactivated in more than half of known human cancers [18]. In order to fully understand the role of p53, it will be crucial to determine its complete set of target genes. The initial p53 matrix (MA0106) comes from JASPAR and was characterized by SELEX [19,20]. The DNA binding site of p53 consensus is a palindrome 'GGACATGCCCGGGCATGTCC' of 20 nucleotides (Figure 4A). The p53 PWM has asymmetrical information content, showing a stronger left half-site. The cytosine at position 6 and the adenine at position 7 are indeed essential for high-affinity DNA binding activity and transactivation [19,20].

542 human p53 ChIP-PET sequences [13] were used as positive sequences, and 1500 sequences from non-coding regions in the human genome *hg*18 assembly as background sequences to optimize the initial p53 matrix from JASPAR. We ran the DISPARE discriminative optimization step and after 5 iterations, the algorithm converged and returned an optimized matrix, referred to as DISPARE matrix (Figure 4B) corresponding to an improved form of the initial PWM refined with the sequence data. The DISPARE matrix has the same length (20 nucleotides) and phase as the initial matrix from JASPAR. The DISPARE matrix recovered the two half sites of p53 and improved the strength of the right half-site.

After optimizing the matrix with DISPARE, the width of the matrix was considered for improvement. The width optimization feature of DISPARE enables the exploration of different lengths (longer or shorter) and phase shifts for the input matrix (see Methods). In this example, we searched all the possibilities of enlarging the input matrix by 0 to 6 nucleotides and performed phase adjustment by trying all possible shifts in 1 nucleotide increment, 3 nucleotides around the matrix position. After running the DISPARE width optimization algorithm, we obtained a new matrix referred to as width-optimized matrix. This matrix has the same length as the initial matrix and the DISPARE matrix, but the phase has been shifted on the right by one column (see Figure 4B). This width-optimized matrix improved the initial JASPAR matrix in two aspects. First, the nucleotide strengths at each position have been refined with regards to the knowledge about p53 binding sites provided by the ChIP-PET data. This is shown by the region between the two half-sites which now look very similar to the matrix derived from the ChIP-PET data in [13]. Furthermore, we gained additional knowledge from the CHIP-PET sequences, particularly the identification of an extra cytosine on the right side of the PWM after the phase optimization, and therefore obtained a matrix with better specificity.

The ROC performances of the DISPARE matrix and the width-optimized matrix are compared to the ones of JASPAR and four selected motif discovery programs: BayesMD, MEME, PREGO and WEEDER (Figure 4C). The motifs returned by these tools were obtained using their default parameters and the search lengths were set according to each program's limitations (see Methods). MEME was used with the -cons option using the consensus 'CCGGACATGCCCGGGCATGT' as prior information. The PWMs from BayesMD, MEME, PREGO and WEEDER were respectively 18, 20, 7 and 12 nucleotides long.

The overall ROC performance of the DISPARE matrix (AUC of 0.98) and the width-optimized matrix (AUC of 0.99) were similar to the ones obtained using JASPAR (AUC of 0.98), BayesMD (AUC of 0.98) and MEME (AUC of 0.98), and outperformed the PREGO and WEEDER matrices which only captured the left half-site. These two *k*-mer based methods were indeed limited to finding motifs of length shorter or equal to 12. Although the AUC of these four matrices are very similar (~0.98), the optimized matrix achieved higher sensitivity than the initial matrix in the [0-0.1] false positive rate interval. The performance increase was small here since the initial matrix had strong information content.

#### Mouse Nanog

**Figure 5:**
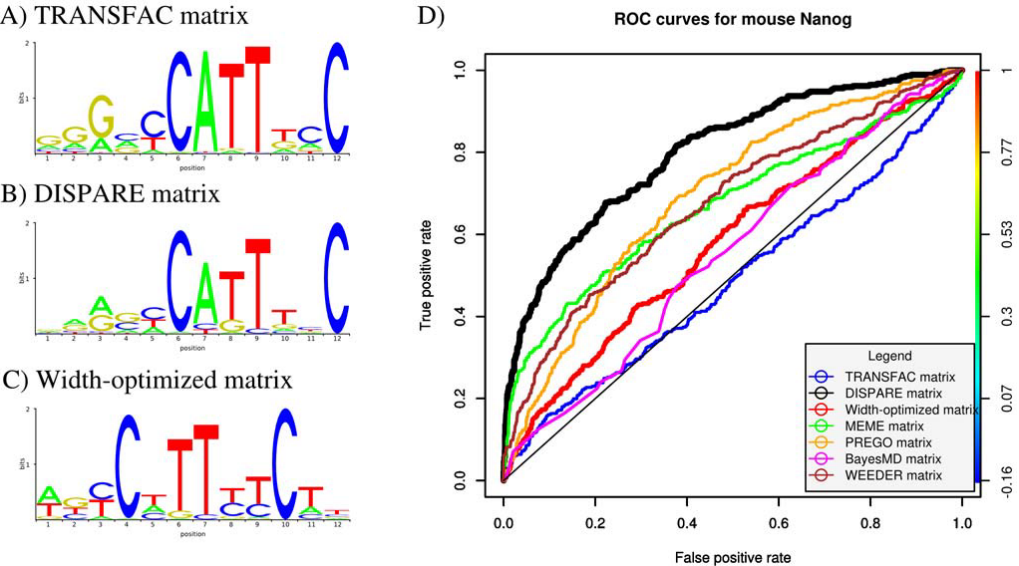
**Refinement of the mouse Nanog matrix (TRANSFAC)**. The sequence logos of the initial TRANSFAC mouse Nanog, DISPARE and width-optimized matrices are shown in A), B) and C). The performances of these three models are compared to those of four motif discovery programs: BayesMD, MEME, PREGO and WEEDER. The results are summarized in the ROC curves shown in D).

Nanog is one of the key TFs that regulates self-renewal and pluripotency in embryonic stem cells. The initial matrix (M01123) comes from TRANSFAC. The motif was identified by NestedMICA [21] in the Nanog ChIP-PET dataset [14] and it is 12 nucleotides long (Figure 5A). We used 376 mouse Nanog ChIP-PET sequences [14] as positive sequences, and 1500 sequences from non-coding regions in the mouse genome *mm*9 assembly as background sequences.

After running the discriminative refinement algorithm on the ChIP-PET dataset for 2 iterations, the algorithm converged and we obtained the DISPARE matrix shown in Figure 5B. The information of the Nanog motif was refined using the ChIP-PET sequences from [14]. After the DISPARE discriminative refinement step, we attempted to obtain a better motif width for the DISPARE matrix. The width optimization step returned a width-optimized matrix of the same length (12 nucleotides) but centered the matrix around the motif core 'CATTTCC'. The information of the width-optimized matrix was changed according to this new width (Figure 5C).

We then compared the ROC performances of the DISPARE matrix and the width-optimized matrix with the ones of the initial TRANSFAC matrix, and matrices obtained with BayesMD, MEME, PREGO and WEEDER (Figure 5D). We ran MEME in supervised mode using the consensus 'CCATTTCC' as prior information. The initial matrix has a poor ROC performance, shown by an area under curve of 0.49. This can be explained by the lack of information in the initial matrix. The low complexity of the Nanog motif can be confused with random background, yielding a high rate of false positives. We observed a significant improvement in the ROC performance for the DISPARE matrix (AUC of 0.80). Furthermore, our algorithm outperformed both *k*-mer based methods: PREGO (AUC of 0.69) and WEEDER (AUC of 0.67), and probabilistic methods: BayesMD (AUC of 0.55) and MEME (AUC of 0.67). Statistical approaches such as MEME, BayesMD and NestedMICA (initial matrix) tend to produce noisier PWMs than *k*-mer constrained approaches, weakening the ROC performance. In this case, the width-optimized matrix (AUC of 0.58) did not improve the performance of the DISPARE matrix. The width optimization algorithm tends to center the matrix around the strongest signal in a motif. However, the Nanog motif consists of two parts, with a weak left side. The proposed new width missed this left side of the motif and was therefore suboptimal compared to the DISPARE matrix. The results of this test demonstrate that the DISPARE algorithm can significantly improve PWM with low information content and represents a good alternative to classical *de novo *motif discovery methods.

#### Human estrogen receptor *α*

**Figure 6:**
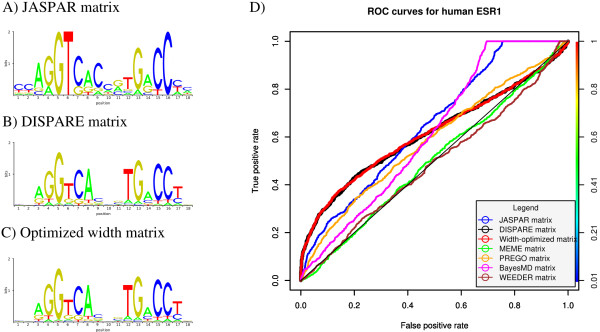
**Refinement of the human ER *α *matrix (JASPAR)**. The sequence logos of the initial JASPAR ER *α*, DISPARE and width-optimized matrices are shown in A), B) and C). The performances of these three models are compared to those of four motif discovery programs: BayesMD, MEME, PREGO and WEEDER. The results are summarized in the ROC curves shown in D).

The estrogen receptor (ER) is a ligand-dependent TF that binds to conserved estrogen response elements (EREs), 5'-GGTCA*nnn*TGACC-3', where *n *is any nucleotide. There are two ER subtypes, ER*α *and ER*β *that belong to the nuclear receptor family. ER*α *whose gene name is ESR1 is over-expressed in *>*70% of breast cancer cases and is likely to be involved in tumorigenesis, although the mechanism underlying this involvement is still unclear [22]. We used 1285 human estrogen receptor *α *binding sites ChIP-PET sequences [15] as positive sequences, and 1500 sequences from non-coding regions in the human genome *hg*18 assembly as background sequences. Running the discriminative optimization algorithm with the JASPAR ESR1 matrix (MA0112) as input (Figure 6A), we obtained a DISPARE matrix after 5 iterations (Figure 6B). The width and phase optimization algorithm returned a width-optimized matrix of the same length and phase as the initial motif as shown in Figure 6C. Both matrices recovered the functional ERE consensus (5'-GGTCA*nnn*TGACC-3') and clearly showed the canonical ERE consensus with a gap of 3 random nucleotides between the two half-sites.

We then compared the ROC performances of the DISPARE matrix and the width-optimized matrix with the ones of the initial JASPAR matrix and matrices obtained with BayesMD, MEME, PREGO and WEEDER (Figure 6D). MEME was used with the -cons option using the consensus 'CCAGGTCACCGT-GACCCC' as prior information. The DISPARE matrix obtained the same results as the BayesMD (AUC of 0.61) and performed performed better than all other methods in the high specificity range (FPR less or equal to 0.5). As the discriminative step of our algorithm returned a DISPARE matrix consisting of high quality matching sites, the ERE element has been over-selected, while flanking (columns 1, 2, and 18) and spacing columns (9, 10 and 11) containing low information have been cleaned. Therefore, as shown on the ROC curve, the DISPARE matrix improved the sensitivity of the motif in the high specificity range (FPR between 0 and 0.4). However, the information that had been removed contributed to the sensitivity when more false positives (FPR between 0.4 and 1) were allowed, explaining the improved behaviour of the initial matrix in that interval. The width and phase optimization step returned a width-optimized matrix with the same width as the DISPARE matrix and therefore showed a similar performance (AUC of 0.62). In this test, we showed that DISPARE enabled us to obtain a better matrix with stronger information in both functional ERE half-sites. We also observed a performance increase in the high specificity range, meaning that the optimized matrices would perform better than the others, using high thresholds.

## Conclusion

Computational characterization of the binding specificity of transcription factors (TFs) is a challenge to the analysis of transcriptional regulation. PWMs are widely used to identify TFBS although they result in a large number of false positives. The general concept of TFBS prediction is to find a set of over-represented sites in a set of sequences of interest compared to a TFBS depleted background set. Many strategies have been proposed, falling into two large categories. Probabilistic approaches are the most widely used methodologies to solve motif discovery problems. The general concept consists of performing a local multiple alignment on the input sequences to build a PWM. Programs such as the Gibbs Sampler [23], NestedMICA [21] or BayesMD [24] use Gibbs sampling to achieve this goal while MEME [25] and a number of other tools use Expectation Maximization. K-mer based methods infer PWMs from sets of statistically significant words that do not differ by more than a certain number of mismatches. These programs include WEEDER [26], PREGO [27] or Scanseq [28]. However, presently available motif discovery algorithms suffer from a lack of sensitivity and specificity, resulting in noisy and incorrectly framed PWMs.

A number of studies have used computational methods to improve PWMs. Bucher (1990) [7] proposed an iterative approach to maximize the over-representation of sites in a window inside a set of aligned promoter sequences enriched with the TFBSs of interest. However, this method assumes that all the subsequences within the preferred window contain functional sites, omitting the problem of false positives. Tsunoda *et al. *(1999) [8] improved the original algorithm by introducing an estimate of the background frequency of sites. Gershenzon *et al. *(2005) [29] proposed a novel approach to improve the sensitivity and specificity of PWMs by maximizing the correlation coefficient instead of the over-representation of sites.

In this paper, we have investigated the refinement of existing matrix models and have focused on the optimization of their width and information content. DISPARE requires a set of positive sequences, a set of background sequences and an initial PWM as input. Our iterative algorithm used a variable threshold for each iteration, so that DISPARE selected subsequences in the original set that scored above the current threshold to build a new matrix. The algorithm stopped after a fixed number of iterations or when the dissimilarity between two consecutive PWMs was smaller than a certain threshold. In the present study, we considered only the maximum scoring occurrence per sequence. In addition, we proposed the optional width and phase optimization step to search a better width for the DISPARE matrix. It returns the best candidate with optimal length and phase. Although changing the phase of the DISPARE matrix can slightly affect the AUC, the width optimization step can also improve the 'biological value' of the matrix as described in the p53 study. In Figure 4, we showed that although the width-optimized matrix and the DISPARE matrix have the same AUC (AUC of 0.99 for the width-optimized matrix and AUC of 0.98 for the DISPARE matrix), the width-optimized matrix is the most biologically interesting solution as it returns a PWM with additional binding information on the column farthest to the right, i.e the extra cytosine that is absent from the original JASPAR model.

The DISPARE algorithm was evaluated on artificial and real datasets in order to measure its performance in both controlled and practical conditions. The simulations on distorted synthetic data demonstrated that the algorithm was able to recover and enhance the matrix of a known TFBS if the matrix was strongly distorted. The test on distorted matrices showed that our algorithm improves the information content of weak motifs, even though the information content is not directly optimized. We used the original and distorted matrices as input in order to evaluate the robustness against noise.

Since known matrices often differ from models that are derived from real datasets, it would have been interesting to repeat the simulation using the original matrix as input to derive the models from the two distorted datasets. Instead, we performed this test on real data. Using ChIP-PET data, we demonstrated that our algorithm optimized the initial matrices, thereby improving the sensitivity and specificity of the initial matrix. Width optimization step can significantly improve the performance of the initial and DISPARE matrices, especially in cases of symmetrical motifs that do not contain strong information on the flanking columns. The choice of an optimal window is not trivial and several solutions can exist. Therefore, the width optimization algorithm can be used in a semi-automated way, by selecting which window to return.

Furthermore, the comparison with four motif discovery tools BayesMD, MEME, PREGO and WEEDER showed, in most cases, that our refined matrices performed better than those derived from these tools. Probabilistic methods were, in general, better than *k*-mer based approaches on real data, achieving performances that were comparable to DISPARE. *K*-mer based approaches were penalized by their motif length limitations since the motifs in the ChIP-PET datasets consisted of 12 or more nucleotides.

Therefore, when the TF is known, the use of a discriminative matrix refinement approach, starting with a known matrix, seems to be more appropriate and offers a good alternative to presently available *de novo *motif discovery methods. In addition, combining motif discovery with matrix refinement into an integrated analysis framework in order to enhance the signal of computationally discovered matrices can significantly improve the results, making matrix annotation and identification easier.

In this study, we presented DISPARE, an algorithm to refine a motif that best discriminates between a positive set of sequences and a background one. Refined matrices are centered, their widths are optimized and they show an improvement in their information content. The algorithm was applied to several sets of synthetic data, distorted data and three sets of ChIP-PET data. The results showed that the DISPARE algorithm improves the sensitivity and specificity of matrix models for TFBSs. This can be used as a good alternative to classical motif scanning methods or combined with *de novo *motif discovery programs. In this version, we only kept one occurrence of a motif, corresponding to the maximum score per sequence and the algorithm returned the most centered and informative refined matrix only. However, because the same transcription factor binding site (TFBS) often occurs in many instances, it would be interesting to compare the final matrices, obtained with several scores per sequence, with those obtained with the maximum score per sequence. In the future, the program can be extended to support multiple motif occurrences and allow the optimization of several windows.

## Methods

### Data

We used three sets of ChIP-PET data: 542 human p53 ChIP-PET sequences [13], 376 mouse Nanog ChIP-PET sequences [14] and 1285 human estrogen receptor (ER) *α *binding sites ChIP-PET sequences [15]. For the background set of sequences, we used 1500 sequences from non-coding regions of the human and mouse reference genomes. All sequences are available on the UCSC genome browser: http://genome.ucsc.edu[30].

### Algorithm

This program refines a PWM to discriminate maximally between a positive and a negative set of sequences. As input, the algorithm uses two sets of sequences and a threshold. There are two sets of sequences: *S*^+^, supposed to contain binding sites of the transcription factor *F*, and *S*^-^, supposed to contain no binding sites of *F*. *S*^+ ^is used as the positive sequences and *S*^- ^as the background sequences. The output is a newly generated PWM. The main steps of the algorithm are scoring all the sequences of positive and negative sets, determining a cutoff, creating a new PWM and, if the end criterion is not satisfied, returning to the scoring step. Finally, either an optimized PWM, referred to as the DISPARE matrix, is obtained, or the initial PWM is retained. The flowchart of the algorithm is described in Additional file 1.

The first step of the algorithm (*score_seq*) consists of scoring all the sequences (*S*^+ ^and *S*^-^) with the current PWM. Only the maximum score per sequence is used. For the first iteration, we chose to start with a threshold *T *= 0, the sequences *S*^+^, the sequences *S*^- ^and the initial PWM. For each iteration, the sequences *S*^+ ^and the sequences *S*^- ^are scanned with the PWM using the current threshold. If a hit of a score is higher than the threshold *T *in the sequences *S*^+^, the score is termed as a true positive. Similarly, if a hit of a score higher than *T *is found in *S*^-^, it is referred as a false positive.

The second step (*get_percentile*) involves determining the cutoff that best separates *S*^+ ^from *S*^-^. In other words, we want to find a threshold that both maximizes the number of matching sequences in *S*^+ ^and minimizes this number in *S*^-^.

For each iteration, a measure of an over-representation between *S*^+ ^and *S*^- ^is calculated for each threshold of the score using a percentile *p*. The percentile value gives the score for which (1 - *p*)% of the scanned sequences do not match the matrix. As we aim to minimize the number of matches in the negative set, the percentile value in *S*^- ^is chosen as the threshold. The threshold value is chosen by the user and can vary. However, we found that *p *= 30 was the value that gave the best compromise when separating *S*^+ ^from *S*^- ^in our datasets. The value of *p *was determined empirically by looking at the number of matches in both sets at different thresholds, while varying values of *p*. Then all the matches in *S*^+ ^above the cutoff were picked to estimate a new PWM.

The third step of the algorithm (*compute_newpwm*) is to make a new matrix with all the matches in *S*^+ ^that scored higher than the threshold *T*. The newly generated matrix has the same width as the initial one. The positional matrix which contains the number of occurrences of a nucleotide at each position is computed. Each column represents a position in the motif and each row corresponds to the number of occurrences of a nucleotide in the motif. From the count matrix, a log-odd matrix is calculated using a uniform background frequency. A pseudo-count value (= 0.1) is added to the count matrix. The log-odd matrix is normalized so that the columns add up to 1 and the log-odd scores are calculated.

The fourth step (*compare_mat*) is to compare the newly generated position frequency matrix (PFM) to the previous one. The Kullback-Leibler Divergence (KLD) [31] or relative entropy measures the difference between two probability distributions. In matrix representations, the binding affinities of a transcription factor are described in each column by independent multinomial probability distributions. Therefore this measure is a natural way to compare PFMs. The Kullback-Leibler divergence is defined as:(4)

where P represents the initial PFM (or the previously generated PFM) and Q represents the newly generated PFM (or the next PFM to be generated).

KLD is always non-negative but in general it is not symmetric. The matrix which provides the smallest value of divergence is the matrix that is most similar to the reference matrix. The KLD is calculated between two consecutive PFMs. The iteration stops before 10 iterations or when KLD ≤ *epsilon*, (that is when the optimized matrix does not change significantly after a given iteration), where *epsilon *is a fixed threshold, e.g 0.001.

After the computation of the new PWM, the next iteration is performed as follows. All the sequences from *S*^+ ^and *S*^- ^are scanned using the newly generated PWM. Steps 2 to 4 are then repeated with the new PWM.

### Width and phase matrix optimization

Determining the correct width of a PWM is an important problem in TFBS characterization. Although programs such as MEME [25], GLAM2 [32] or NestedMICA [21] sample motifs of variable lengths, many computational approaches focus instead on identifying and locating motifs. The width matrix optimization problem includes two aspects: width and phase optimization.

The length of a PWM can be adjusted by either adding or removing one or several flanking columns of the matrix. Finding the optimal shorter matrix is intended to compact the matrix to its most informative part by removing non-informative flanking columns. Thus, the part of the matrix that contributes the most to the total information content, i.e core motif can be identified. However, in real studies, it is often more interesting to look at the nucleotides surrounding actual TFBSs and therefore extend the length of a PWM. The DNA that surrounds TFBSs defines the context in which a site is implanted and may also contribute to the binding. Therefore, picking up these extra nucleotides surrounding TFBSs can improve the performance of a PWM and reduce the number of false positives.

Another important problem in width optimization is the phase shift. Two PWMs with the same length can perform very differently depending on their phase. The goal is therefore to determine the phase shift that achieves the best performance for a given PWM.

In motif discovery studies, PWMs for known TFBSs, or computationally predicted TFBSs, often have a non-optimal frame or window, i.e wrong positioning of the window with regards to the actual biological binding site and non optimal length. Non-optimal framing can be caused by either the addition of extra flanking columns in database matrix models, e.g. two columns in JASPAR, or using experimental or computational TFBS characterization methods that returned a wrong shift of the optimal binding site. In order to handle these sub-optimal matrices, DISPARE provides a width optimization feature that determines the best width and phase shift, if any, after the discriminative optimization step.

The matrix width optimization procedure takes a DISPARE matrix, the optimized matrix by our algorithm, as input and returns a width-optimized matrix, if any, and improved information content. The algorithm consists of two steps: i) width and phase optimization and ii) matrix enhancement according to the new width.

In the width and phase optimization step, the algorithm first explores all the possible ways of adjusting the length of the DISPARE matrix. Matrices are generated in a combinatorial manner by exploring all possibilities of adding or removing *n *columns from the original matrix. For example, increasing the length of the initial matrix by two can be done by adding two columns on the left or two columns on the right or one on each side of the matrix. The nucleotide counts that are needed for matrix enlargements are taken from the sequences flanking the sites in the original matrix.

The new lengths are defined within an interval, for example, ± 6 nucleotides around the length of the input. For the CHIP-PET data, we chose to explore the ways of increasing the width of the DISPARE optimized matrix by 0 to 6 columns, as we wanted to know whether there was additional information about the binding sites outside the original width of the input matrix.

After generating all the matrix length combinations, the algorithm computes all possible phase shifts of the newly generated matrices by shifting the matrix from 0 to 3 nucleotides from the original phase. These two systematic matrix computation steps provide the candidates from which the best phase and shift are chosen.

The selection of the optimal width is done by ranking, based on the information content and centering of the matrices. For each matrix, the average information per column is evaluated. The highest average information value per column corresponds to the most compact matrix. The candidate matrices are first ranked based on this value. In the second step, the algorithm evaluates the centering of each matrix by calculating the distance between the barycenter, i.e position around which the information is centered, and the position of the middle column. The best ranking matrix corresponds to the best centered. Finally, the selected width is the one that achieves the best cumulated rank, ensuring that the optimal matrix is the most informative and well-centered.

After the width optimization step, the optimized matrix undergoes a refinement process that gradually maximizes the information according to the newly selected width. The algorithm follows the same column-wise procedure as described in [33]. In the count-matrix variation of this optimization method, each column is tuned by transferring random fractions of counts from a source to a destination nucleotide. The matrix is scanned over the set of positive sites and the change that achieves the highest score is selected. A column is considered to be optimal if no improvements are observed after a fixed number of random transfers (e.g., 10). The score is defined here as the total log-score for the positive dataset.

### ROC curves

To evaluate the performance of different classifiers, it is routine to use Receiver Operating Characteristic (ROC) graphs [34,35]. In a ROC graph, the sensitivity of a classifier is plotted against (1 - specificity) as the discrimination threshold is varied. Equivalently, the ROC curve can be obtained by representing the rate of true positives (TPR) versus the rate of false positives (FPR).

Accuracy is measured by the area under curve (AUC): an area of 1 represents the best classifier (all true positives are found) and an area of 0 the worst possible classifier (no true positives are found). The more the curve is situated towards the top left, the better the classifier is.

We assessed the performance of the DISPARE matrix refinement algorithm by comparing our findings with those of four *de novo *motif discovery programs: BayesMD [24], MEME [25], PREGO [27] and WEEDER [26]. Motif predictions were performed on each dataset using the default parameters of each program (see Additional file 4). MEME was used with the -cons option. Since the selected motif discovery methods consist of very different design approaches (statistical, *k*-mer based, use of background sequences, etc), data preprocessing (e.g, masking, use of evolutionary conservation tracks, etc) was not allowed.

The effectiveness of the initial matrix, the DISPARE, the width-optimized matrix and the matrices predicted by the four *de novo *programs were evaluated on test and background sequences for the synthetic, p53, Nanog and ESR1 datasets. We used the ROCR package [36] to compute the ROC curves as the true positive rate (TPR) versus the false positive rate (FPR) of each matrix model on the test data.

For the synthetic data, we were able to evaluate the real fitness of the different matrices as we knew the positions of the motifs in each sequence. Our planted hits are defined as "true positives" (TP) while any other hits are considered as "false positives" (FP). A tolerance of 30% (4 nucleotides for matrix *wm *and 2 nucleotides for matrix *wma*) was applied to allow sites to occur within a certain interval around the exact position of the implanted sites. For the ChIP-PET data, we considered the motif that had the highest score for each sequence as a "true positive" and any other scores as "false positives". We also assumed that the background sequences contained no motifs.

## Competing interests

The authors declare that they have no competing interests.

## Authors' contributions

I.P implemented the DISPARE algorithm (except the width matrix optimization part) proposed in the paper, did the data analysis and drafted the manuscript. M.H.T implemented the width matrix optimization, produced the data on width matrix optimization and helped with the draft. O.E conceived and supervised the research plan. All authors read and approved the final manuscript.

## Supplementary Material

Additional file 1 **Figure S1**. Flowchart of the DISPARE algorithm. Click here for file

Additional file 2 **Figure S2**. ROC curves for synthetic data: matrix wma. 5 datasets. Click here for file

Additional file 3 **Figure S3**. ROC curves for synthetic data: matrix wm. 5 datasets. Click here for file

Additional file 4 **Supplementary Material**. Supplementary material. Click here for file
