# Supplementary figures and images for: DISPARE: DIScriminative PAttern REfinement for Position Weight Matrices
Source: BMC Bioinformatics. 2009 Nov 26;10:388. doi: 10.1186/1471-2105-10-388 (PMC2788558; doi:10.1186/1471-2105-10-388)

**Additional file 1 - Figure S1 : Flowchart of the DISPARE algorithm.**

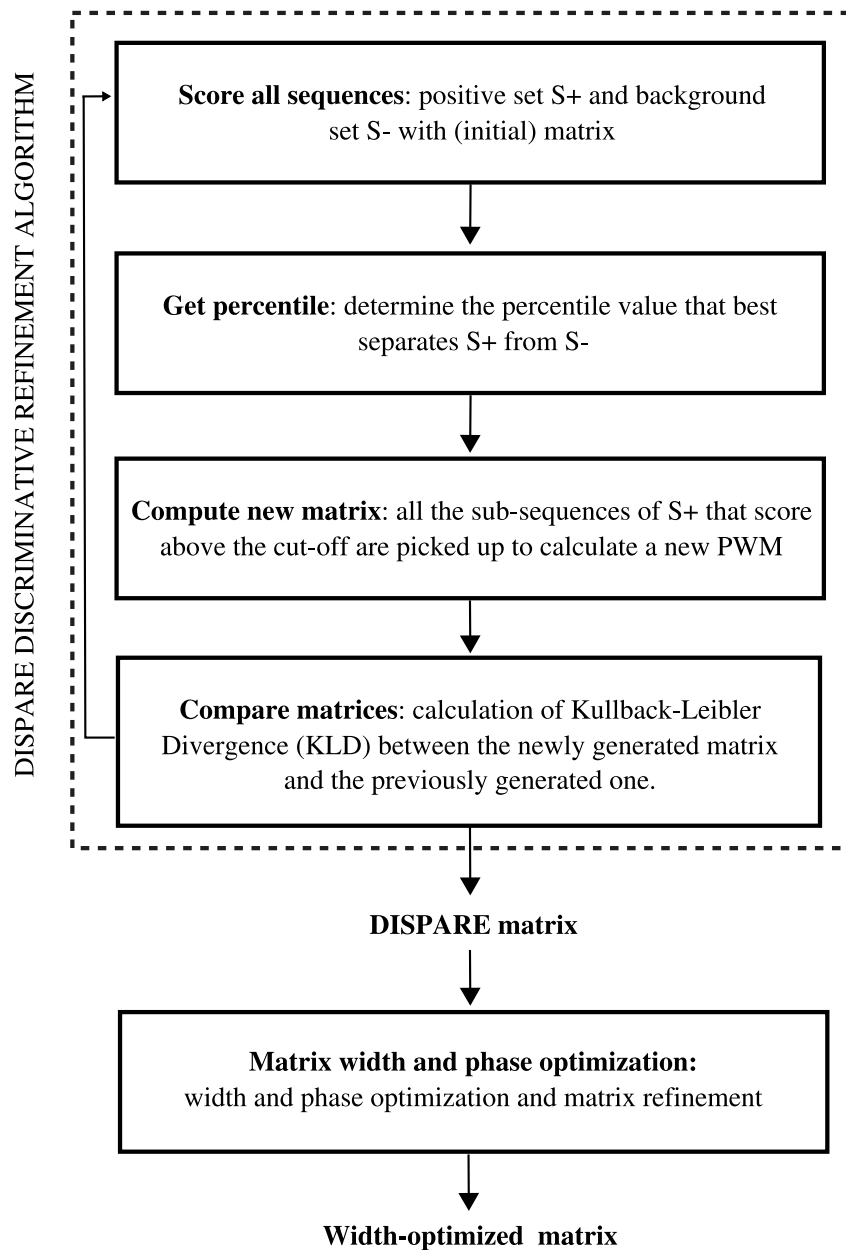

Supplement: Additional file 1 — Figure S1. Flowchart of the DISPARE algorithm. [file 1471-2105-10-388-S1.PDF]

Additional file 2 - Figure S2: ROC curves for synthetic data: matrix wma, 5 datasets.

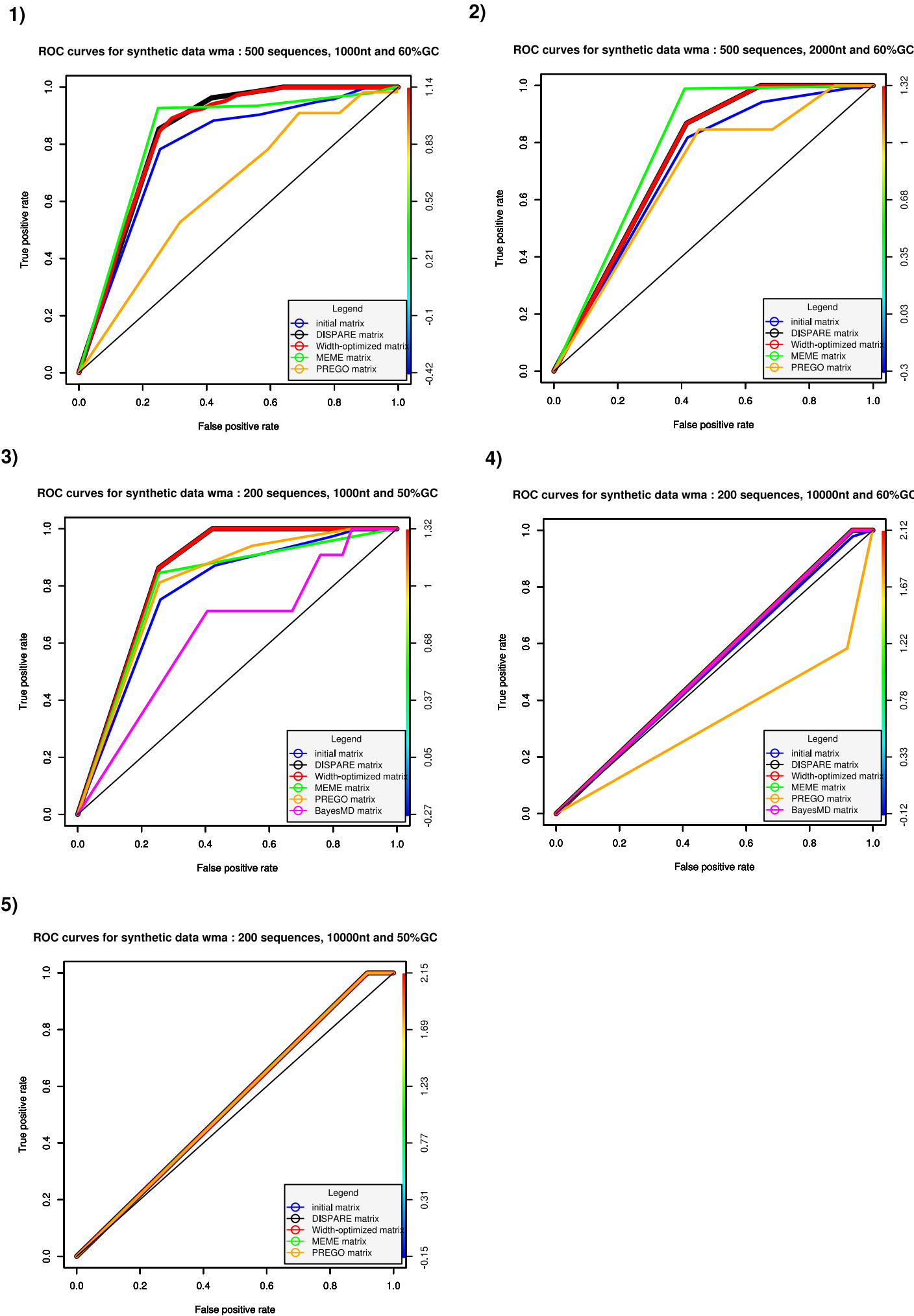

Supplement: Additional file 2 — Figure S2. ROC curves for synthetic data: matrix wma. 5 datasets. [file 1471-2105-10-388-S2.PDF]

# Additional file 3 - Figure S3: ROC curves for synthetic data: matrix wm, 5 datasets.

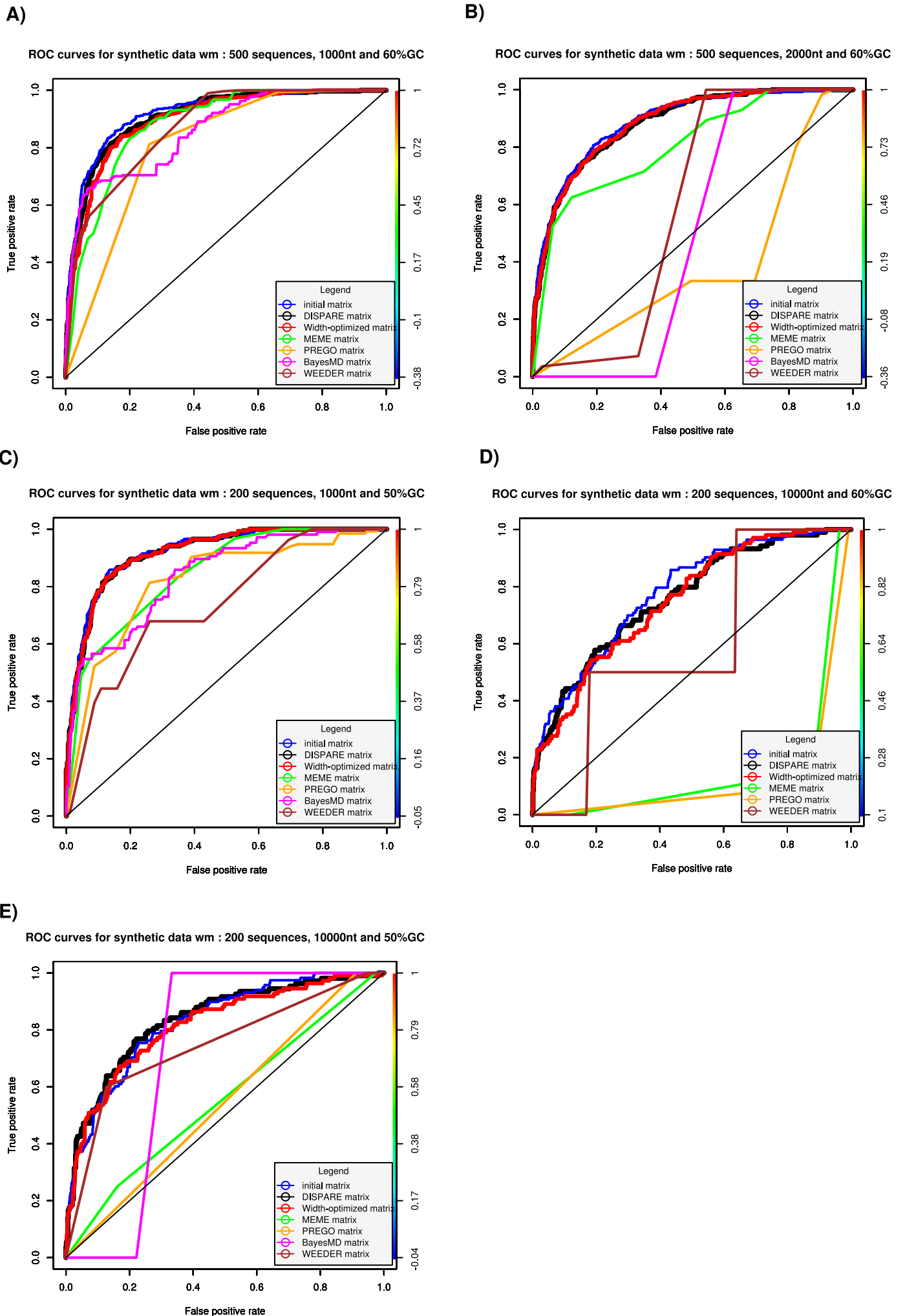

Supplement: Additional file 3 — Figure S3. ROC curves for synthetic data: matrix wm. 5 datasets. [file 1471-2105-10-388-S3.PDF]
