# Supplementary material for: DISPARE: DIScriminative PAttern REfinement for Position Weight Matrices
Source: BMC Bioinformatics. 2009 Nov 26;10:388. doi: 10.1186/1471-2105-10-388 (PMC2788558; doi:10.1186/1471-2105-10-388)
Supplement: Additional file 4 — Supplementary Material. Supplementary material. [file 1471-2105-10-388-S4.PDF]

Additional file 4: Supplementary material for the article

Motif discovery programs

To compare our results, we selected four motif discovery programs from the literature, two probabilistic (BayesMD [1] and MEME [2]) and two *k*-mer-based algorithms (PREGO [3] and WEEDER [4]). In the following we provide a short description of each program and detail the parameters that we used to run the motif predictions.

BayesMD

BayesMD [1] is a probabilistic, Bayesian model for predicting novel transcription factor binding sites. Biological information about binding sites properties, background sequence models, occurrence and positional preferences are built into the model in modular fashion. Mixture prior parameters for the motif and background are trained using information on TFBSs and organism-specific promoter sequences from available databases. The main feature of this method is the use of a positional prior that provides *a priori* information about the location and number of occurrences of the sought motifs.
In our study, the BayesMD program was run on its default parameters (2nd order mixture background) and without informative positional priors, since we decided not to any mapping of data. The motif length was provided as input (w=8 for synthetic and Nanog and w=18 for p53 and ESR1).

MEME

MEME [2] is a probabilistic, EM-based approach to predict novel transcription factor binding sites. In our study, we used the default parameters for the priors (Dirichlet), background (order 0) and set the occurrence model to *zoops* (zero or one occurrences). The motif width was provided as input accordingly to the one of the sought patterns. MEME was tested in both supervised and non supervised mode (data not shown). In the tests with synthetic data, we provided the sought consensus a prior information using the option *-cons*. For the biological data, we tested MEME in its *de novo* motif finder settings. MEME was able to recover the synthetic, p53 and ESR1 matrices without supervision but failed to find the Nanog matrix, which had low information content. Therefore, we forced the convergence by providing the sought consensus 'CCATTTTC' as input.

PREGO

PREGO [3] is an algorithm that performs PWM regression on raw ChIP-on-Chip data. The method consists of two main steps: seed discovery and PWM regression.

In the first step, the program evaluates the statistical significance of all *k*-mer occurrences, (k<9 by default) to determine the seeds that will be used for PWM regression. The p-value calculation is based on a correlation factor between the ChIP raw binding ratio and the number of *k*-mer occurrences.

In our study, raw binding ratio for the input sequences was not available. Therefore, we created a binary expression file that labeled '1' all the positive sequences and '0' the background sequences (see sequence section) and we added the option ' -SuppressLogWarning=1' to handle non logarithmic expression files. Because the seed discovery step consists in an exhaustive analysis of all statistically significant *k*-mers, the program could search patterns of width less or equal to 12 nucleotides. PREGO was able to recover the synthetic, p53 and Nanog matrices in unsupervised mode but failed to find the ESR1 matrix. Therefore we skipped the seed discovery step and forced the program to perform PWM regression for the seed 'GGTCACCGTGA' only.

WEEDER

WEEDER [4] is a *de novo* motif discovery program that evaluates the statistical significance of all *k*-mers of length ranging between 6 and 12. Interesting PWMs are obtained from the *k*-mers by compiling the best sites that match the consensus up to a certain number of mismatches. WEEDER provides an additional program, called adviser that suggests the best PWM candidates for the input sequence data.

References

[1]. Tang MH, Krogh A, Winther O: BayesMD: Flexible biological modeling for motif discovery. J Comput Biol. 2008, Dec;15(10):1347-63.
[2]. Bailey TL, Elkan C: Fitting a mixture model by expectation maximization to discover motifs in biopolymers. Proceedings of the Second International Conference on Intelligent Systems for Molecular Biology,AAAI Press, Menlo Park, California 1994, :28-36.
[3]. Tanay A: Extensive low-affinity transcriptional interactions in the yeast genome. Genome Res. 2006, Aug;16(8):962-72. Epub 2006 Jun 29.
[4]. Pavesi G, Mauri G, Pesole G: An algorithm for finding signals of unknown length in unaligned DNA sequences. Bioinformatics 2001, 17 (Suppl.):207-214.
